# Reduced treatment frequencies with bumped kinase inhibitor 1369 are effective against porcine cystoisosporosis

**DOI:** 10.1016/j.ijpddr.2020.08.005

**Published:** 2020-08-21

**Authors:** Aruna Shrestha, Bärbel Ruttkowski, Patricia Greber, Grant R. Whitman, Matthew A. Hulverson, Ryan Choi, Samantha A. Michaels, Kayode K. Ojo, Wesley C. Van Voorhis, Anja Joachim

**Affiliations:** a Institute of Parasitology, Department of Pathobiology, University of Veterinary Medicine, Veterinärplatz 1, A-1210, Vienna, Austria; b Center for Emerging and Reemerging Infectious Diseases, Division of Allergy and Infectious Diseases, Department of Medicine, University of Washington, 750 Republican Street, Seattle, WA, 98109, USA; c Departments of Microbiology and Global Health, University of Washington, 750 Republican Street, Seattle, WA, 98109, USA

**Keywords:** *Isospora suis*, Oocyst excretion, CDPK1, Growth inhibition, dpi, days post-infection, SD, study day, IPEC, intestinal porcine epithelial cells, BKI, bumped kinase inhibitor, FS, fecal score, AF, autofluorescence, DMSO, dimethyl-sulfoxide, BW, body weight, LoQ, limit of quantitation, LoD, limit of detection

## Abstract

Bumped kinase inhibitors (BKIs) are a new class of antiprotozoal drugs that target calcium-dependent protein kinase 1 (CDPK1) in various apicomplexan parasites. A multiple dose regimen of BKI 1369 has been shown to be highly effective against *Cystoisospora suis* (syn*. Isospora suis*), the causative agent of neonatal porcine coccidiosis. However, multiple dosing may not be widely applicable in the field. The present study aimed to determine the efficacy of reduced treatment frequencies with BKI 1369 against porcine cystoisosporosis *in vitro* and *in vivo*. Pre-incubation of sporozoites with BKI 1369 completely failed to inhibit the infection *in vitro* unless treatment was prolonged post-infection. Notably, a single treatment of infected cell cultures 2 days post-infection (dpi) resulted in a significant reduction of merozoite replication. In an experimental infection model, treatment of suckling piglets experimentally infected with *C. suis* 2 and 4 dpi with 20 mg BKI 1369/kg body weight completely suppressed oocyst excretion. A single treatment on the day of infection or 2 dpi suppressed oocyst excretion in 50% and 82% of the piglets and reduced the quantitative excretion in those that shed oocysts by 95.2% and 98.4%, respectively. Moreover, a significant increase in body weight gain and reduced number of diarrhea days were observed in BKI 1369 treated piglets compared to the control piglets, irrespective of time points and frequencies of treatment. Given that reduced treatment frequencies with BKI 1369 are comparable in efficacy to repeated applications without any adverse effects, this could be considered as a practical therapeutic alternative against porcine cystoisosporosis.

## Highlights

• BKI 1369 does not target host cell invasion by *C. suis* sporozoites but replication of merozoites.
• Single treatment with BKI 1369 in parallel with experimental infection is not effective for the control of cystoisosporosis*.*
• Two doses of BKI 1369 at 2 and 4 dpi completely suppressed oocyst excretion in piglets experimentally infected with *C. suis.*

## Graphical abstract

**Figure:**
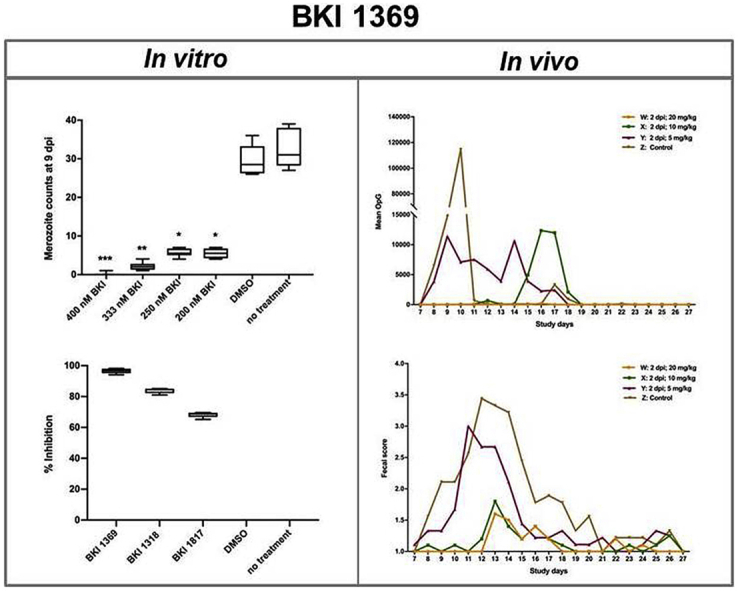

## 1 Background

Cystoisosporosis is one of the major diarrheal diseases in neonatal piglets caused by the apicomplexan parasite *Cystoisospora suis* (syn. *Isospora suis* (Barta et al., 2005)). Infection during the first days after birth is associated with pasty-to-watery non-hemorrhagic diarrhea, unthriftiness and poor weight gain (Joachim and Shrestha, 2020). The disease is generally non-fatal but shows high morbidity in affected litters (Joachim and Shrestha, 2020; Lindsay et al., 1985). It has a wide prevalence (Hinney et al., 2020; Joachim and Shrestha, 2020) thereby inducing substantial economic losses in the pig farming industry (Kreiner et al., 2011; Mundt et al., 2007; Skampardonis et al., 2010). Current therapeutics for cystoisosporosis are limited to the triazinone toltrazuril (Mehlhorn and Greif, 2016). However, a toltrazuril-resistant *C. suis* strain has recently been reported (Shrestha et al., 2017) and emergence of widespread resistance against anticoccidials, as described for avian coccidia (Chapman, 1984; Stephan et al., 1997) cannot be ruled out. Moreover, a potential hazard of toltrazuril residues is also of concern, a reason for which Canada and the USA did not approve Baycox® for use in pigs (Government of Canada, 2005), leaving swine farmers without effective treatment.

Bumped kinase inhibitors (BKIs) are promising drugs against apicomplexan parasites, including *C. suis* (Choi et al., 2020; Doggett et al., 2014; Hulverson et al., 2017a; Ojo et al., 2016; Ojo et al., 2014; Shrestha et al., 2019). BKIs are a group of competitive inhibitors of ATP-binding that selectively target apicomplexan calcium-dependent protein kinases (CDPKs) without affecting mammalian kinases (Choi et al., 2020; Keyloun et al., 2014). CDPKs are crucial for multiple physiological functions in apicomplexan parasites such as gliding, cell invasion, egress and replication (Ojo et al., 2012). These CDPKs are absent in mammalian hosts and can be selectively targeted by BKIs (Keyloun et al., 2014). Multiple doses of BKI 1369 have previously been shown to effectively reduce oocyst excretion and diarrhea and consequently improve body weight gain in piglets experimentally infected with *C. suis*, without adverse effects (Shrestha et al., 2019). However, the previously tested BKI 1369 treatment regimen required treating each piglet twice a day for five days, which is not practicable in the field due to increased labor costs and handling stress for the piglets. Therefore, the present study aimed to evaluate efficacy of reduced treatment frequencies with BKI 1369 against cystoisosporosis. Given that only infection within first few days of life leads to significant disease outcome and oocyst shedding, and older piglets exhibit age-related resistance (Joachim and Shrestha, 2020), we hypothesized that the treatment efficacy can be maintained during the critical life stage of the piglets with reduced frequencies of drug application. Moreover, we sought to identify the most appropriate time point of treatment as well as the minimal effective drug concentration required to develop a practical drug application scheme for the prevention of suckling piglet cystoisosporosis.

## 2 Materials and methods

### 2.1 Compounds

BKI 1369 and its metabolites, BKI 1318 and BKI 1817, were synthesized by VAS Bio, Cherlaplly, Hyderabad, India, as described previously (Johnson et al., 2012; Shrestha et al., 2019; Vidadala et al., 2016) and were >95% pure, as determined by high performance liquid chromatography.

### 2.2 *C. suis* strain propagation and *in vitro* cultivation

The laboratory strain *C. suis* Wien I (Joachim and Mundt, 2011), was maintained by passage in suckling piglets every 3–6 months and infectivity *in vivo* was assessed regularly at the Institute of Parasitology, University of Veterinary Medicine, Vienna. Oocysts were purified from collected feces, brought to sporulation and stored as described elsewhere (Worliczek et al., 2013). For *in vitro* studies, sporozoites were excysted (Worliczek et al., 2013), enumerated with a hemocytometer and used immediately for infection of semi-permanent intestinal porcine epithelial cells (IPEC-1). For *in vivo* studies, sporulated oocysts were washed with tap water and enumerated prior to infection of piglets.

### 2.3 *In vitro* experiments

IPEC-1 cells were maintained in culture medium at 37 °C and 5% CO_2_ (DHEM/HAM12 supplemented with 5% fetal calf serum and penicillin/streptomycin; Gibco via Thermo Fisher, Vienna, Austria). Cells (4 × 10^4^) were used to seed 24-well plates and grown to confluence. All BKI compounds were stored as 20 mM stock solutions in 100% dimethyl sulfoxide (DMSO) at −20 °C. Cell viability in the presence of DMSO and BKIs was determined by colorimetric cell proliferation (WST-1) assays (Shrestha et al., 2019). In addition IPEC-1 cells were infected with serial dilutions of *C. suis* sporozoites, obtained after excystation to evaluate the growth curves of merozoites during 5–9 days of cultivation. To determine the effect of BKI 1369 on parasite replication, sporozoites (5 × 10^2^) were added to each well one day after seeding of host cells and incubated with different concentrations of BKI 1369 at 40 °C for various time periods or a DMSO control, in triplicate.

In order to test whether BKI 1369 affects initial host cell invasion by *C. suis*, freshly excysted sporozoites were pre-incubated with BKI 1369 or DMSO for 6 h at 37 °C. Subsequently, sporozoites were washed with medium and used for infection of cell monolayers (5 × 10^2^/well), in triplicate. Readout for both growth (merozoite replication) and invasion-inhibition experiments was microscopical evaluation by counting free merozoites 9 days post-infection (dpi) from culture supernatants. Supernatants were collected and free merozoites were counted in pooled samples at 9 dpi from quadruplicate wells in C-Chip disposable hemocytometers (NanoEnTek/Roth Lactan, Graz, Austria). All assays included DMSO and no-treatment controls in quadruplicate.

### 2.4 Study animals

Two separate animal experiments were conducted. In each of the experiments, conventionally raised healthy piglets from three crossbred sows (Landrace x Large White) were randomly allocated to four treatment groups (Table 1). Two weeks before farrowing, the sows were transferred to the animal facility of the Institute of Parasitology, University of Veterinary Medicine Vienna, Austria to acclimatize to the housing conditions. Sows were housed on straw in individual farrowing crates and fed once daily with a commercial feed free of coccidiostats. All rooms were equipped with daylight and ventilation, and a room temperature of 18–20 °C was maintained throughout the trials. The piglets received milk from the sow followed by starter feed from the second week of life. Fresh drinking water was provided ad libitum to the sows and piglets. The first day after the birth of piglets was considered as study day (SD) 1. All piglets were ear-tagged and received 200 mg iron dextran on SD 2 to prevent iron deficiency anemia. The clinical study lasted for 29 days (SD 29) and followed a blinded and randomized experimental block design with the individual piglet as an experimental unit.

**Table 1:** Litters and treatment groups in experiment I and II; piglets were infected on study day (SD) 3; dpi: day post-infection.

Groups	Litter No.	No. of piglets enrolled	No. of piglets that completed the study^a^	Treatment dose (mg/kg of body weight)	Day of treatment
Experiment I					
**A**	1,2,3	10	10	1 × 20	SD 3 (0 dpi)
**B**	1,2,3	11	11	1 × 20	SD 5 (2 dpi)
**C**	1,2,3	11	11	2 × 20	SD 5 & SD 7 (2 & 4 dpi)
**Control-D**	1,2,3	11	10^a^	1 x mock^c^	SD 3
Experiment II					
**W**	1,2,3	10	10	1 × 20	SD 5 (2 dpi)
**X**	1,2,3	10	10	1 × 10	SD 5 (2 dpi)
**Y**	1,2,3	9	9	1 × 5	SD 5 (2 dpi)
**Control-Z**	1,2,3	10	9^b^	1 x mock^c^	SD 5

a **One piglet from control group was found dead on SD 11. Only those piglets that completed the study were included in the analysis of efficacy.**

b **One piglet in the control group was found dead on SD 20. Only those piglets that completed the study were included in the analysis of efficacy.**

c **Mock: solvent (3% Tween 80 + 7% ethanol + 90% 0.9% NaCl)**.

All the procedures involving animals were approved by the Animal Ethics Committee of the University of Veterinary Medicine Vienna and the national authority according to § 26ff of Animal Experiments Act, Tierversuchsgesetz 2012-TVG 2012 (license number: BMWF-68.205/0034-WF/V/3b/2016; Austrian Federal Ministry of Science, Health and Economy).

### 2.5 Experimental infection

On SD 3, each piglet was orally infected with 1000 sporulated oocysts of *C. suis* (strain Wien-I) suspended in 1 ml tap water using a flexible plastic Pasteur pipette.

### 2.6 Treatments

Fine powder of BKI 1369 was dissolved in solvent (3% Tween 80 + 7% ethanol + 90% normal saline) to yield 5%, 2.5% and 1.25% solutions. In experiment I, all piglets except those in the control group, were treated with 20 mg/kg body weight (BW) BKI 1369 (0.4 ml 5% BKI 1369/kg BW) on the day of infection (Group A, single dose), 2 dpi (Group B, single dose) or 2 and 4 dpi (Group C, two doses). In experiment II, piglets in groups W, X and Y were treated 2 dpi with a single dose of 20 mg/kg BW (0.4 ml 5% BKI 1369/kg BW), 10 mg/kg BW (0.4 ml 2.5% BKI 1369/kg BW) and 5 mg/kg BW (0.4 ml 1.25% of BKI 1369/kg BW) BKI 1369, respectively. Piglets in the control groups (Groups D and Z) were mock treated with the solvent only (Table 1).

### 2.7 Post-treatment observations

For both experiments, post-treatment observations for any adverse events such as swelling/bleeding of the iron injection site, inability to stand, walk, suckle or other abnormal behavior, including dyspnoea, vomiting, limping, lateral recumbency, signs of pain, distress or neurological alterations (Oldham, 1985), were conducted under blinded conditions by a veterinarian at 2 h, 24 h and 48 h after treatment. In addition, all piglets were observed daily during the course of the studies to ensure good health, and any condition that required veterinary intervention was recorded and addressed.

### 2.8 Sample collection and efficacy parameters

The efficacy of BKI 1369 was evaluated by the determination of parasitological and clinical parameters. Individual fecal samples were collected daily from SD 7 to 27 for the evaluation of oocyst excretion and fecal consistency. Fecal samples were screened for the presence of oocysts by autofluorescence (AF) (Daugschies et al., 2001), and positive samples were further quantified with a modified McMaster technique (Joachim et al., 2018). Fecal consistency was scored immediately after sampling with fecal scores (FS) 1: normal/firm, FS 2: pasty, FS 3: semi-liquid and FS 4: liquid; FS 3 and 4 were considered as diarrhea. To evaluate the presence of other entero-pathogens causing diarrhea, a pooled fecal sample from each litter was collected on the first day of sampling (SD 7) and screened for the presence of rotavirus, coronavirus, *Escherichia coli*, and *Clostridium perfringens*. The body weight of each piglet was recorded on SD 1, 8, 15, 22, 29 to determine body weight development and additionally on the day/s of treatment for calculation of the treatment dose/s.

Additionally, to determine exposure to BKI 1369 and its metabolites, BKI 1318 and BKI 1817, pooled fecal samples per group were collected before treatment (−24 h), 2, 24 and 48 h after treatment and at the end of the study (SD 29; experiment I). Approximately 1 g of feces was soaked overnight in 3 ml phosphate buffered saline (PBS, pH 7.4). The following day, 12 ml of acetonitrile containing 100 μM propanolol as an internal standard was added and the mixture was thoroughly vortexed and centrifuged at 1000×*g* for 30 min. Clear supernatant was then transferred to Eppendorf tubes and concentrated in a miVac® Duo Concentrator (SP Scientific, Pennsylvania, USA) at 35 °C. Control/blank samples spiked with known amounts of target analyte were processed in tandem to provide a standard curve. The concentrated samples were shipped to the University of Washington and were stored at −20 °C until analysis by liquid chromatography-tandem mass spectrometry (LC-MS/MS) to determine fecal pharmacokinetics. All LC-MS/MS analyses were carried out on a Waters Acquity UPLC coupled with a Xevo TQS Micro and analyzed using MassLynx software (Waters Corporation, Milford, MA, USA). Extraction and LC-MS/MS conditions are described in detail in the Supplementary File S1.

In experiment II, additional fecal samples were collected from piglets which shed oocysts after treatment with BKI 1369. Fecal DNA preparation was carried out by established procedures. PCR assays were used for the amplification of the genomic DNA fragments encoding *Cs*CDPK1. PCR assays used primers upstream (166 and 91 base pairs of the start codon) and downstream (89 and 39 base pairs) of the *Cs*CDPK1 stop codon fragment to generate templates for a subsequent nested run. Nested PCR amplification of the 5955 base pair fragment was achieved using the primer pair LIC_*Cs*CDPK_Fwd: GGGTCCTGGTTCGATGGGGCAGCAAGAGAGTGTTC and LIC_*Cs*CDPK_Rev: CTTGTTCGTGCTGTTTATTATCGGTTTCCACAGAGCTTCAAAAG. PCR amplicons were cloned into TA TOPO 2 vectors (Invitrogen, Carlsbad, California, USA) and sequenced at GENEWIZ Inc. (Seattle, USA) to confirm the presence or absence of mutations that could affect drug sensitivity. Moreover, two piglets from each group were sacrificed at the end of the study (SD 29; 24 days after treatment) and tissue samples (liver, muscle, kidney, backfat, jejunum) were collected and processed similar to the fecal samples in experiment I, except that 20 nM propranolol was used as an internal standard instead of 100 μM, to determine residues of BKI 1369 and its metabolites.

### 2.9 Statistical analyses

Statistical calculations for *in vivo* trials were performed with RStudio version 1.1.453 (RStudio Team, 2018), descriptive statistics with Microsoft Excel 2010 (Microsoft Inc., Vienna, Austria) and GraphPad Prism version 5.04 for Windows (GraphPad Software, San Diego, California USA). Differences in clinical and parasitological parameters between groups were analyzed with ANOVA in the case of normal distribution and variance homogeneity of the data, or a Kruskal-Wallis rank sum test if this was not the case. In the event of significance for the omnibus tests, parametric or non-parametric *post-hoc* tests for multiple comparisons were performed (according to Tukey and Conover, respectively), with *P*-value adjustment after Bonferroni. *In vitro* assays were evaluated by t-tests with significance reported at *P* < 0.05. For testing of efficacy *in vitro*, the percentage of growth/invasion inhibition was calculated as follows:

$$\%\;inhibition\;=\;(1-\;\frac{\text{No. of merozoites counted at }9\text{ dpi in BKI }1369\text{ treated culture }}{\text{No. of merozoites counted at }9\text{ dpi in DMSO}-\text{only treated culture}\;})\;x\;100$$

## 3 Results

### 3.1 *In vitro* experiments

**Fig. 1:**
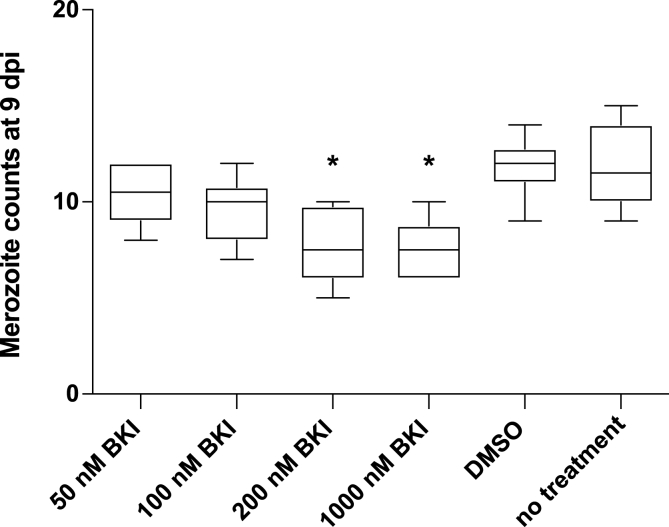
Inhibition of host-cell invasion ability of sporozoites pre-incubated in different concentrations of BKI 1369 at 37 °C *in vitro*; asterisk indicate significant differences *P ≤ 0.05.

**Fig. 2:**
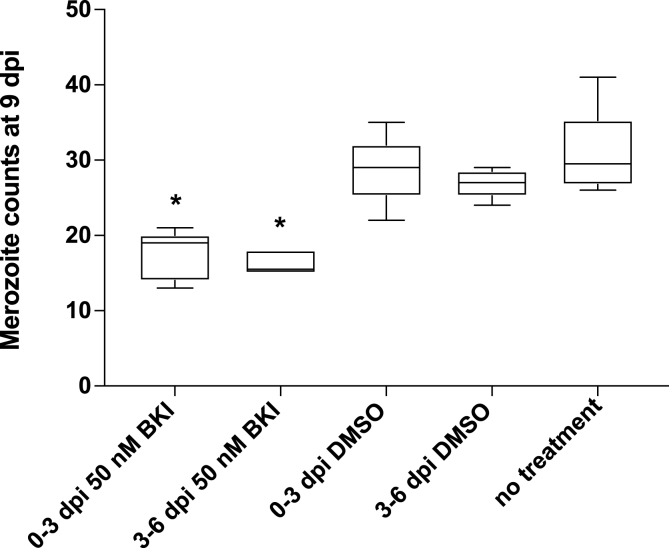
Comparison of efficacy of 50 nM BKI 1369 applied for 0–3 *vs* 3–6 days post-infection *in vitro*; asterisk indicate significant differences *P ≤ 0.05.

**Fig. 3:**
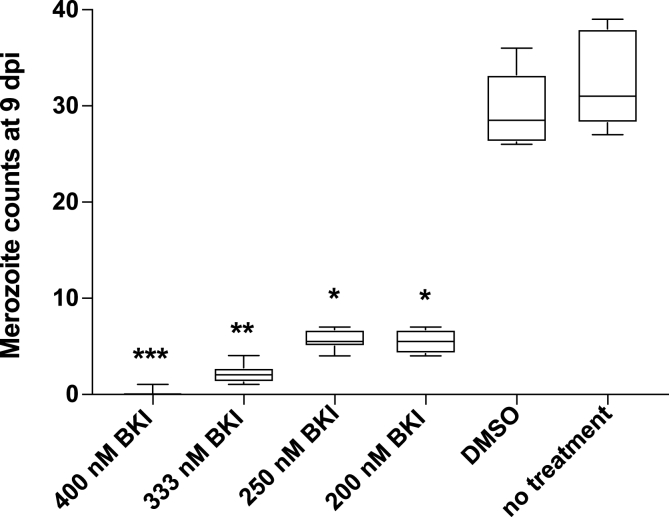
Inhibition of merozoite replication by single doses of BKI 1369 at different concentrations applied for one day (2 days post-infection); asterisks indicate significant differences *P ≤ 0.05, **P ≤ 0.01 and ***P ≤ 0.001.

Pre-incubation of sporozoites with concentrations of BKI 1369 ≥ 200 nM significantly reduced the total merozoite counts at 9 dpi (*P* < 0.05), whereas concentrations ≤100 nM completely failed to inhibit host cell invasion compared to DMSO-only and no-treatment controls (Fig. 1). In a further experiment conducted to determine the effects of treatment in relation to the time-point after infection, incubation of infected host cells with 50 nM BKI 1369 at 0–3 dpi or at 3–6 dpi significantly inhibited merozoite replication at 9 dpi compared to that of DMSO-only and no-treatment controls (*P* < 0.05) (Fig. 2). Incubation of infected host cells with a single application of BKI 1369 2 dpi resulted in a significant reduction of merozoite replication at 9 dpi for concentrations of ≥200 nM or higher (Fig. 3)*.* Lastly, treatment of infected host cells with 400 nM of BKI 1369 and its metabolites, BKI 1318 and 1817 once at 2 dpi decreased the total merozoite counts by 96.1%, 85.1% and 69.6%, respectively compared to the DMSO-only and no-treatment controls (Supplementary Figure S1).

### 3.2 Animal experiments I & II

#### 3.2.1 Oocyst excretion

**Fig. 4:**
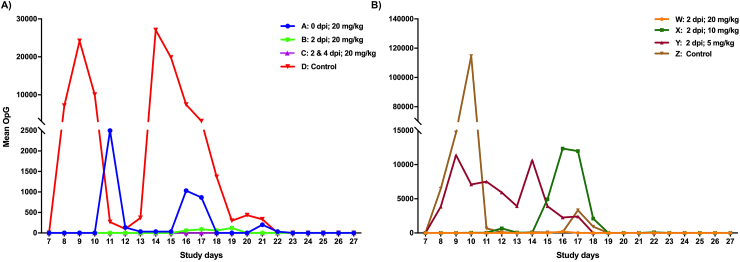
Mean oocysts per gram of feces during the whole study period as determined by modified McMaster technique. (A) Effect of 20 mg BKI 1369/kg BW administered at different time points; (B) Effect of different concentrations of BKI 1369 administered 2 days post-infection. BW: body weight.

Excretion of oocysts started on SD 8 in both control groups. All piglets in the control groups, except one in group Z, excreted oocysts at least once. Mean duration of oocyst excretion was 4.4 ± 2.9 days in group D and 3.3 ± 1.5 days in group Z. In experiment I, oocyst excretion was completely suppressed by two doses of 20 mg BKI 1369/kg BW at 2 and 4 dpi (group C), while two piglets in group B, which had received 20 mg BKI 1369/kg BW at 2 dpi, excreted oocysts starting on SD 16 (Fig. 4A). In contrast, 50% of piglets in group A (which had received 20 mg/kg BW on the day of infection) excreted oocysts. In this group, excretion started on SD 11 with a mean duration of 1 ± 1.6 days (Supplementary Table S1). All parameters related to oocyst excretion such as percentage of days with McMaster detectable oocyst excretion, the mean number of oocysts per gram of feces (OpG) and the area under the curve (AUC) for OpG were significantly different between the treated and control groups. In addition, AUC for OpG and the number of excretion days were significantly different between group A (treatment on the day of infection) and group C (two treatments at 2 and 4 dpi) (Table 2).

**Table 2:** P values (given as –log10) for parameters of oocyst excretion, fecal score and body weight gain. Differences at *P* < 0.05 are indicated in bold. OpG: oocysts per grams of feces; FS: fecal score.

Experiment I	Groups					
Parameter	A *vs* D	B *vs* D	C *vs* D	A *vs* C	B *vs* C	A *vs* B
Area under the curve for OpG	**4.23**	**7.35**	**8.77**	**1.85**	0.00	0.66
Number of excretion days	**4.02**	**6.55**	**8.23**	**1.54**	0.00	0.27
Area under the curve for FS	0.43	**2.92**	**2.20**	0.56	0.03	1.02
Number of diarrhea days	0.66	**3.74**	**3.17**	0.60	0.00	1.04
Daily weight gain	**1.62**	**1.35**	**1.66**	0.00	0.00	0.00
Total weight gain	**1.62**	**1.35**	**1.66**	0.00	0.00	0.00

**Experiment II**	**Groups**					
**Parameter**	**W *vs* Z**	**X *vs* Z**	**Y *vs* Z**	**W *vs* Y**	**X *vs* Y**	**W *vs* X**
Area under the curve for OpG	**4.76**	**2.47**	0.00	**4.61**	**2.33**	0.36
Number of excretion days	**4.45**	2.08	0.00	**4.79**	**2.40**	0.42
Area under the curve for FS	**4.82**	**3.67**	0.00	**2.86**	**1.88**	0.00
Number of diarrhea days	**4.82**	**3.56**	0.19	**2.63**	**1.47**	0.00
Daily weight gain	**3.92**	**3.22**	**1.88**	0.40	0.14	0.03
Total weight gain	**3.92**	**3.22**	**1.88**	0.40	0.14	0.03

In experiment II, oocyst excretion was effectively suppressed by a single dose of 20 mg BKI 1369/kg BW (group W), except for one piglet which excreted oocysts only once on SD 16 (Fig. 4B). In contrast, 50% of piglets in group X, which received 10 mg BKI 1369/kg BW, and 90% of piglets in group Y, which received 5 mg BKI 1369/kg BW, excreted oocysts with a mean duration of 1.3 ± 2.7 and 3.6 ± 1.9 days, respectively (Supplementary Table S1). The area under the curve for OpG was significantly lower in groups W and X compared to the control group Z and group Y. Compared to the control group Z, the number of excretion days was significantly lower only in group W, while both groups W and X had significantly lower number of excretion days compared to group Y (Table 2).

### 3.3 Fecal score

**Fig. 5:**
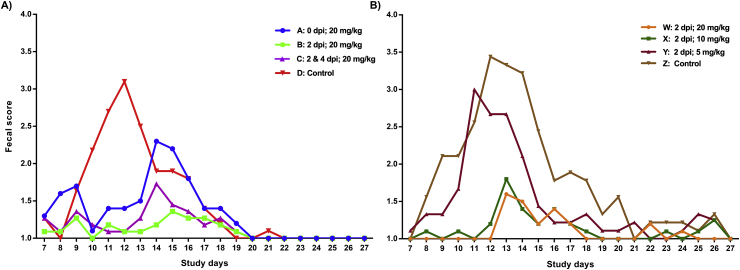
Mean fecal score of piglets throughout the sampling period of 21 days. (A) Effect of 20 mg BKI 1369/kg BW administered at different time points; (B) Effect of different concentrations of BKI 1369 administered 2 days post-infection. BW: body weight.

In both experiments, the maximum prevalence of diarrhea was 90% in the control groups with an average duration of 3–4 days. None of the piglets had diarrhea on the day of infection. In experiment I, piglets that received a single dose at 2 dpi or two doses at 2 and 4 dpi had significantly lower mean FS and fewer diarrhea days compared to the control piglets (Fig. 5A, Table 1). In experiment II, treatment at 2 dpi with BKI 1369 in doses of 10 mg and 20 mg/kg BW effectively suppressed diarrhea, while a dose of 5 mg/kg BW completely failed to control diarrhea in infected piglets (Fig. 5B, Table 2). AUC of FS and number of days with diarrhea were also significantly different between the group that had received 5 mg/kg BW and the two groups with higher treatment doses (Table 2).

### 3.4 Body weight development

**Fig. 6:**
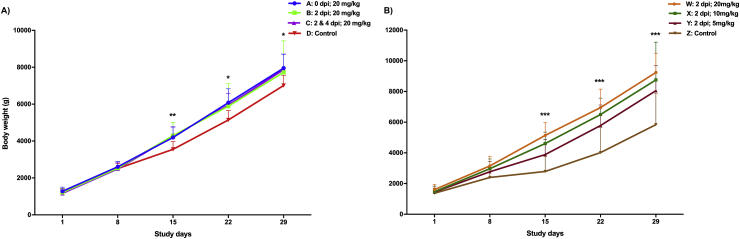
Body weight development in piglets after BKI 1369 treatment. (A) Effect of 20 mg BKI 1369/kg BW administered at different time points; (B) Effect of different concentrations of BKI 1369 administered 2 days post-infection. Vertical lines depict standard deviations, asterisks indicate significant differences at different weighing days of *P ≤ 0.05, **P ≤ 0.01 and ***P ≤ 0.001. BW: body weight.

In both experiments, body weights were not significantly different between groups on SD 1 (day of randomization) and SD 8 (Fig. 6A and B). While no differences were found among BKI treated groups in either experiment, daily weight gain and total body weight gain from SD 1 to 29 were significantly higher in the treated groups compared to the control group in both experiments (Fig. 6A and B; Table 2). During the acute phase of infection SD 8 to 15, control groups in both experiments had a severe depression of weight gain; the mean body weight gain of piglets in the control group D was 1042 g compared to 1594 g, 1762 g and 1773 g in treated groups A, B and C, respectively. Similarly, in experiment II, the mean BW of piglets in control group Z was 389 g during the acute phase of infection compared to 1992 g, 1638 g and 1120 g in treated groups W, X and Y, respectively (Supplementary Table S2).

### 3.5 Differential diagnosis

Fecal samples pooled by litters on SD 7 were negative for rotavirus and coronavirus, whereas *E. coli* and *Cl. Perfringens* could be detected in all litters in both experiments.

### 3.6 Safety

No piglets showed treatment-related adverse effects that required veterinary intervention throughout the study period. Neither visible signs of toxicity nor clinically relevant systemic or regional/local responses related to treatment were observed in the treated groups.

### 3.7 Fecal drug kinetics

Fecal concentrations of BKI 1369 and its metabolites BKI 1318 and BKI 1817 were analyzed by LC-MS/MS as a proxy for intestinal exposure. The maximum fecal BKI 1369 concentration (C_max_) and the time at which that maximum concentration was reached (T_max_) were determined.

Pooled feces from group A (single treatment with 20 mg BKI 1369/kg on SD 3, day of infection) were collected at −24, 2, 24, 48, and 624 h post-dose. BKI 1369 T_max_ was observed at 24 h post dose with a C_max_ of 8.1 μM. BKI 1318 T_max_ was observed at 48 h post-dose with a C_max_ of 0.4 μM. BKI 1817 T_max_ was observed at 48 h post-dose with a C_max_ of 60.8 μM (Table 3).

**Table 3:** BKI concentrations in pooled fecal samples by treatment groups. Piglets were infected on SD 3; SD: study days; dpi: days post-infection.

			Group A (20 mg BKI 1369/kg BW; 0 dpi)			
			Concentration (μM)			
SD		Hours post first-dose	BKI 1369	BKI 1318	BKI 1817	
**2**		**−24**	0.0	0.0	0.0	
**3**		**2**	0.8	0.0	0.0	
**4**		**24**	8.1	0.3	54.0	
**5**		**48**	5.5	0.4	60.8	
**29**		**624**	0.0	0.0	0.0	
**Group B (20 mg BKI 1369/kg BW; 2 dpi)**						
**4**	**−24**		0.0	0.0	0.0	
**5**	**2**		0.0	0.0	0.0	
**6**	**24**		0.8	0.0	0.8	
**7**	**48**		3.0	0.7	53.5	
**29**	**576**		0.0	0.0	0.0	
**Group C (20 mg BKI 1369/kg BW; 2 and 4 dpi)**						
**4**		**−24**	0.0	0.0		0.0
**5**		**2**	0.0	0.0		0.0
**6**		**24**	4.5	0.3		130.0
**7**		**48/2 h post 2**nd **dose**	37.0	1.9		280.8
**8**		**72/24 h post 2**nd **dose**	19.7	3.6		331.6
**9**		**96/48 h post 2**nd **dose**	29.2	7.0		537.0
**29**		**576/528 h post 2**nd **dose**	0.0	0.3		6.6

Pooled feces from Group B (single treatment with 20 mg BKI 1369/kg on SD 5) were collected at −24, 2, 24, 48, and 576 h post-dose. BKI 1369 T_max_ was observed at 48 h post-dose with a C_max_ of 3.0 μM. BKI 1318 T_max_ was observed at 48 h post-dose with a C_max_ of 0.7 μM. BKI 1817 T_max_ was observed at 48 h post-dose with a C_max_ of 58.5 μM (Table 3).

Pooled feces from Group C (multiple treatments with 20 mg BKI 1369/kg on SD 5 and SD 7) were collected at −24, 2, 24, 48, 72, 96, and 576 h post first dose. BKI 1369 T_max_ was observed at 48 h post first-dose with a C_max_ of 36.9 μM. A second peak 48 h after the second dose was also observed with a lower concentration of 29.1 μM. BKI 1318 T_max_ was observed at 96 h post first-dose with a C_max_ of 7.0 μM. This metabolite continued to build and did not exhibit a dual peak as did the parent BKI 1369. BKI 1817 T_max_ was observed at 96 h post-dose with a C_max_ of 537 μM. Similar to BKI 1318, this metabolite continued to build and did not exhibit a dual peak (Table 3).

### 3.8 Tissue residues

At the end of experiment II, six piglets (two piglets each from BKI 1369 treated groups W, X and Y) were sacrificed 24 days after treatment and samples of liver, kidney, muscle, backfat and jejunum tissues were isolated for compound residual analysis of BKI 1369 and its metabolites. All tissue types from treated piglets had BKI 1369 and metabolite levels below the limit of quantitation (LoQ), except for muscle and jejunal samples of one piglet from group X which received 10 mg BKI 1369/kg BW 2 dpi (muscle: BKI 1369 0.09 μM; BKI, 1817 0.17 μM; jejunum: BKI, 1817 0.11 μM). The LoQ varied by BKI and tissue type, but was generally 0.08 μM or less, except for BKI 1318 in the jejunum with a LoQ of 0.2 μM. Due to background signal in the blank samples, a limit of detection (LoD) was not readily ascertained. Some additional sample levels were above the background level but below the LoQ, indicating that in at least some samples, BKI 1369, BKI 1318, and BKI 1817 are still present in the tissue at relatively low levels. Data detailing the LoQ and samples that ranged between the signal background and LoQ for each tissue type can be found in Supplementary Table S3.

### 3.9 DNA sequence analysis

To determine whether oocysts that are excreted after BKI 1369 treatment have acquired resistance mutations in *Cs*CDPK1, the target of BKI 1369, we isolated the oocysts, extracted DNA and used nested PCR to sequence *Cs*CDPK1. DNA sequences were analyzed using online tools (BLAST and CDART) available at the National Center for Biotechnology Information. *Cs*CDPK1 genomic DNA was characterized by PCR and sequencing experiments. Only two (samples 603 and 508) of the seven samples yielded a negative amplification reaction with nested primer pairs targeting the whole coding region. In the five samples that could be analyzed, there were no nucleotide base mutations within the ATP binding domain where BKI 1369 binds to *Cs*CDPK1.

### 3.10 Discussion

Bumped kinase inhibitors possess selective and specific anti-parasitic properties against a wide range of apicomplexan parasites of human and veterinary importance (Hulverson et al., 2017a). Apicomplexans that utilize glycine as a gatekeeper residue in the ATP binding site of CDPK1, such as *Toxoplasma, Cryptosporidium, Neospora*, and *C. suis*, are specifically sensitive to BKIs (Hulverson et al., 2017a; Van Voorhis et al., 2019). They block synthesis of CDPK1 which plays important roles in various signal transduction cascades in the parasite's life-cycle, such as host cell attachment and invasion, sexual differentiation, gliding motion, egress etc. (Billker et al., 2009; Kugelstadt et al., 2011; Ojo et al., 2012). Previously, we have shown that treatment for five days with ten doses of BKI 1369, a known inhibitor of *Cs*CDPK1, significantly suppressed clinical disease in experimentally infected piglets (Shrestha et al., 2019). In the present study we evaluated more practical approaches of applying BKI 1369 with reduced treatment frequencies *in vitro* and *in vivo*, as well as a dose titration experiment with a single treatment post-infection.

In *Toxoplasma*, BKIs are known to inhibit host cell invasion by sporozoites by suppressing *Tg*CDPK1, which is essential during invasion (Kieschnick et al., 2001; Lourido et al., 2012). Being a close relative, *C. suis* was expected to be similarly affected by BKIs. Incubation of *C. suis* sporozoites with BKI 1369 before infection resulted in significant reductions in parasite replication only at BKI concentrations ≥200 nM *in vitro*. These results suggest that molecular effects of BKI 1369 are not limited to inhibition of initial host cell invasion by sporozoites. Similar to the previous report of redundancy of CDPK1 during erythrocytic invasion in *P. berghei* (Jebiwott et al., 2013), *Cs*CDPK1 function might be dispensable during host cell invasion by *C. suis* sporozoites, although further studies are warranted.

Moreover, similar results of treatment with 50 nM BKI 1369 at 0–3 dpi and at 3–6 dpi indicated that BKI 1369 has antiparasitic effects during asexual endogenous development of *C. suis*. This assumption was further supported by the fact that a single treatment of infected host cells at 2 dpi with ≥200 nM BKI 1369 resulted in a significant reduction of the total merozoite counts at 9 dpi. In addition, it was noteworthy that repeated treatment was not required to significantly reduce the parasite burden *in vitro* compared to the no-treatment and DMSO controls.

Although major guidelines for drug efficacy studies are routinely established under stringent *in vitro* conditions, high variability in the clinical environment is expected (Bi et al., 2009). Therefore, two animal experiments were conducted applying an established piglet infection model to translate *in vitro* results obtained so far to *in vivo* applications. The treatment regime adopted in the previously published study (Shrestha et al., 2019) was tedious, demanding considerable time and workforce for animal handling. Apart from being effective in controlling disease, an ideal drug for farm animals should also ensure minimal animal handling, thereby reducing the stress of drug administration (Stuart and Robinson, 2015) as well as costs of labor. Therefore, in the present study, efficacy of single or double oral doses of BKI 1369 administered at different time points were tested.

BKI 1369 given at 20 mg/kg BW 2 dpi or 2 and 4 dpi significantly reduced oocyst excretion and diarrhea and improved body weight gain. In contrast, a single dose of 20 mg BKI 1369/kg BW administered on the day of infection failed to reduce occurrence of diarrhea in infected piglets, although oocyst shedding was minimized compared to the control group. This result is consistent with the *in vitro* results of this study, further confirming that BKI 1369 has a better efficacy when administered 2 dpi, targeting merozoites, compared to treatment on the day of infection, targeting sporozoites. Based on these results, treatment as early as on the 3rd day of life would be recommendable under field conditions, so that BKI 1369 is effective even if piglets contract infection immediately after birth, considering high infection pressure in a highly contaminated environment. Since *C. suis* has short prepatency (4–6 days (Joachim and Shrestha, 2020; Shrestha et al., 2015),), BKI treatment within this period largely prevents oocyst excretion. In older pigs, natural age-resistance prevents disease development with no or less oocyst shedding (Koudela and Kučerová, 1999), thereby, further validating the importance of treatment during prepatency.

Inclusion of BKI 1369 as a therapy into current clinical practice requires determination of the minimum effective dose, which not only ensures effective control of the parasitic infection, but also limits drug residues in animal products. A single application of 5 mg BKI 1369/kg BW 2 dpi completely failed to control parasitic shedding and diarrhea. By contrast, higher single doses, 10 and 20 mg BKI 1369/kg BW, applied at the same time point, significantly suppressed oocyst excretion and diarrhea compared to the control group, indicating that the minimal effective dose of BKI 1369 against cystoisosporosis is 10 mg/kg BW for single treatment. However, although not statistically significant, more piglets that received a single dose of 10 mg BKI 1369/kg BW (5 out of 10) excreted oocysts for longer periods, thereby increasing infection pressure for other piglets via environmental oocyst contamination compared to a single positive sample in the group that received a single dose of 20 mg BKI 1369/kg BW. Since no mutations were observed in the *Cs*CDPK genes isolated from *C. suis* positive fecal samples after BKI 1369 treatment, the continued excretion *of C. suis* DNA is not likely due to acquired resistance but more likely related to a sub-optimal treatment regimen.

All tissue samples isolated 24 days after treatment (except the muscle and jejunum samples from a single piglet from group X) were below the LoQ for BKI 1369 and its metabolites. However; due to noise in the blank samples, a true LoD could not be achieved. In previous experiments with whole (un-dried) piglet samples (Shrestha et al., 2019), a similar signal noise was observed in blank samples, suggesting that piglet samples inherently give a variable background signal using our established extraction and LC/MS-MS detection methods rather than cross-contamination during the drying process. Only one piglet had quantifiable levels of BKI 1369 and metabolites, and values were below 0.2 μM for all tissue types. While other piglets had detectable levels, they were all below the LoQ, indicating that there is considerable inter-individual variability with respect to metabolism and excretion. With such a small sample size, and only a single time point at SD 29, it is difficult to come to a meaningful conclusion regarding tissue residues and how these persist or are cleared in pigs. Additional studies looking at different sacrifice dates, increasing the sample size, and more sensitive detection methods are warranted to address this issue.

Drug exposure at the exact localization of parasites within their host is crucial to the development of novel therapeutics (Arnold et al., 2017). The previous study reported BKI 1369 levels in plasma at different time points after treatment. However, given that the majority of the life cycle of *C. suis* takes place in intestinal epithelial cells, systemic plasma exposure may not be the best correlate for *in vivo* efficacy required for controlling parasite replication. For BKIs, a correlation between fecal BKI concentrations and *in vivo* efficacy has been reported earlier (Hulverson et al., 2017b). Therefore, in the present study, fecal BKI concentrations were measured assuming that this reflects gut/gut enterocyte levels of BKI 1369 better than plasma concentrations. Group B showed the lowest C_max_ of parent and metabolites and the slowest improvement in clinical response, further indicating the importance of intestinal exposure to compound for effective suppression of intestinal stages of apicomplexan parasites. In general, BKI 1369 peaked within 24–48 h whereas the metabolite levels continued to rise beyond 48 h. In all samples, levels were below the LoQ in the feces at SD 29. BKI 1817 appears to be the major metabolite excreted in the feces, whereas BKI 1318 appears to have relatively low excretion via the feces.

## 4 Conclusion

In addition to the evaluation of therapeutic efficacy, pharmacokinetics and safety parameters of reduced treatment frequencies, the present study also evaluated the minimum effective concentration of BKI 1369 required to combat porcine cystoisosporosis. A single dose of 10 mg BKI 1369/kg BW was highly effective in reducing oocyst shedding and diarrhea, and improved body weight gain in infected piglets, therefore it can be considered as the minimal effective dose to control *C. suis* infections. It should be combined with thorough cleaning and disinfection to minimize the infection pressure (Hinney et al., 2020; Joachim and Shrestha, 2020). Treatment of piglets at 3 days of life is recommended to receive best treatment efficacy, since BKI 1369 targets merozoites, not the invasive sporozoites, and should be applied after infection but before the onset of oocyst excretion and diarrhea for maximum efficacy. Since reduced treatment frequencies with BKI 1369 are comparable in efficacy to repeated applications without any adverse effects, this could be considered as a practical therapeutic alternative against porcine cystoisosporosis.

## Declarations

### Ethics approval

All the procedures involving animals were approved by the Animal Ethics Committee of the University of Veterinary Medicine Vienna and the national authority according to § 26ff of Animal Experiments Act, Tierversuchsgesetz 2012-TVG 2012 (license number: BMWF-68.205/0034-WF/V/3b/2016; Austrian Federal Ministry of Science, Health and Economy).

## Consent for publication

Not applicable.

Availability of data and materials.

All data and materials of the experiments described here are included in the manuscript and its additional file.

## Funding

Research studies reported in this publication were partially funded by 10.13039/100014851Bayer Animal Health, project number FA16119038; 10.13039/100000060National Institute of Allergy and Infectious Diseases (NIAID, 10.13039/100000002National Institutes of Health (NIH), 10.13039/100011408USA under award numbers R01AI089441, R21AI123690, R21AI140881, R01AI111341 and R01HD080670. This study was also supported by award number 2014-06183 from the United States 10.13039/100000199Department of Agriculture (USDA)/10.13039/100005825National Institute of Food and Agriculture (NIFA).

## Author contributions

AS, AJ and WVV conceived and designed the study. AS performed the animal experiment, analyzed the samples and carried out statistical analyses. BR carried out *in vitro* experiments. GRW conducted LC-MS/MS analysis on tissue samples and fecal samples. KKO and SAM performed PCR and sequence analysis of fecal DNA. AS drafted the manuscript. All authors read and approved the final manuscript.

## Declaration of competing interest

Dr. Wesley C. Van Voorhis is an officer and owns stock in ParaTheraTech Inc., a company that is trying to bring BKIs to the animal health market. He helped to design the experiments and edited the paper, but did not have a role in performing or interpreting the results. Ryan Choi, Matthew A. Hulverson, and Grant R. Whitman have been compensated for prior consulting work done for ParaTheraTech Inc.

## Appendix A Supplementary data

Supplementary Figure S1 Inhibition of development of C. suis merozoites by a single dose of 400 nM BKI 1369 and its metabolites BKI 1318 and BKI 1817 at 2 days post-infection *in vitro*. Supplementary Figure S1

Supplementary Table S1 Overview of parasitological and clinical parameters in all experimental groups. AUC: area under the curve; OpG: oocysts per grams of feces; SD: standard deviation. Supplementary Table S1

Supplementary Table S2 Overview of body weight development (in grams) of piglets in different groups with standard deviations in brackets. SD: study day, BW: body weight, BWG: body weight gain. Supplementary Table S2

Supplementary Table S3 Limit of Quantitation (LoQ) for BKI 1369 by tissue type. Summary of samples above the LoQ and those that fall between background signal and the LoQ. Tissue samples were taken on study day 29. Supplementary Table S3

Supplementary File S1 Compound detection and quantification by LC-MS/MS. Supplementary File S1
