# Supplementary material for: Reduced treatment frequencies with bumped kinase inhibitor 1369 are effective against porcine cystoisosporosis
Source: Int J Parasitol Drugs Drug Resist. 2020 Aug 21;14:37–45. doi: 10.1016/j.ijpddr.2020.08.005 (PMC7442133; doi:10.1016/j.ijpddr.2020.08.005)
Supplement: Supplementary Figure S1 — Inhibition of development of C. suis merozoites by a single dose of 400 nM BKI 1369 and its metabolites BKI 1318 and BKI 1817 at 2 days post-infection in vitro. [file mmc1.docx]

Tissue and feces from vehicle control treated piglets were used as blanks or standards. Calibration curves for BKI 1369, BKI 1318, and BKI 1817 were prepared by adding 2 μL BKI in dimethylsulfoxide (DMSO) to 18 μL tissue or feces controls to generate nominal concentrations of 0.004, 0.008, 0.02, 0.04, 0.08, 0.2, 0.4, 0.8, 2, 5, 10, and 20 μM. Calibration curves were generated separately for each tissue type and feces.

5 μL of tissue homogenate curves and samples were added to tubes that contained 95 μL of 80:20 acetonitrile: water. As an internal standard, propranolol was added to each sample to a final concentration of 20 nM. Samples were dried to completion using a miVac Duo Concentrator (SP Scientific, Pennsylvania, USA) at 35 °C. Sample processing for feces was similar but utilized a slightly different process to concentrate the sample as explained in the methods. Dried Samples were sealed and shipped to the University of Washington for LC-MS/MS analysis.

The day prior to analysis, dried samples were reconstituted in 100 μL of acetonitrile, vortexed for 15 seconds and allowed to sit at ambient temperature for 24 h. The following day, samples were plated on Nunc™ 96-Well Polypropylene Storage Microplates (Thermo Fischer Scientific™, Rockford, IL, USA), mixed vigorously on a bench top shaker at for 15 min and subsequently centrifuged at 4150 x *g* for 10 min. Supernatant from each sample was placed in a new 96-well plate, sealed, and analyzed by LC-MS/MS. All samples from the pharmacokinetic/residue experiments were measured with an Acquity UPLC in tandem with a Waters Xevo TQ-S micro and analyzed using MassLynx software (Waters Corporation, Milford, MA, USA). The solvents were A: water with 0.1% formic acid and B: acetonitrile with 0.1% formic acid. Sample concentrations were determined using internal standard normalized calibration curves. All analyses were run with electrospray ionization in positive ionization mode and solvent gradient at curve of 6: 0 min 95%A, 5%B; 1 min 95%A, 5%B; 5 min 5%A, 95%B; 6.5 min 5%A, 95%B; 6.6 min 95%A 5%B; 8 min 95%A 5%B. BKIs 1369, 1318, and 1817 analytes were monitored and quantified with the m/z transitions 418.2🡪112.0, 404.2🡪97.6, and 390.1🡪112.0 respectively. Propranolol was used as an internal standard and monitored with the m/z transition 260.0🡪127.3.
