# Supplementary figures and images for: Reduced treatment frequencies with bumped kinase inhibitor 1369 are effective against porcine cystoisosporosis
Source: Int J Parasitol Drugs Drug Resist. 2020 Aug 21;14:37–45. doi: 10.1016/j.ijpddr.2020.08.005 (PMC7442133; doi:10.1016/j.ijpddr.2020.08.005)

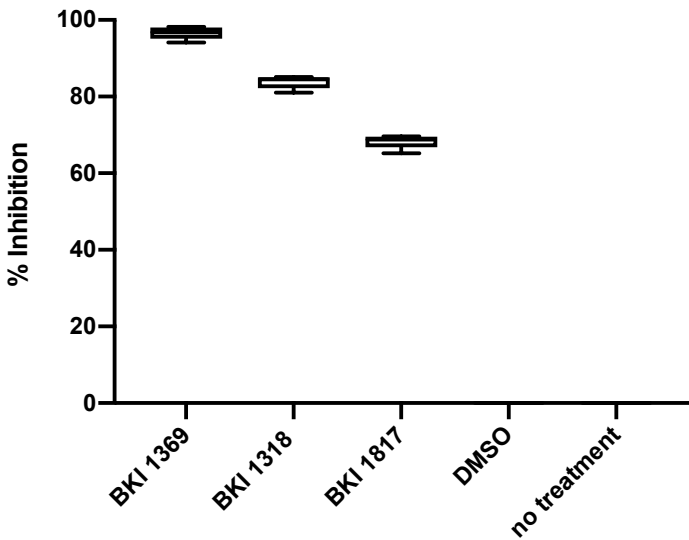

Supplement: Supplementary Table S1 — Overview of parasitological and clinical parameters in all experimental groups. AUC: area under the curve; OpG: oocysts per grams of feces; SD: standard deviation. [file mmc2.pdf]
