# Supplementary material for: Reduced treatment frequencies with bumped kinase inhibitor 1369 are effective against porcine cystoisosporosis
Source: Int J Parasitol Drugs Drug Resist. 2020 Aug 21;14:37–45. doi: 10.1016/j.ijpddr.2020.08.005 (PMC7442133; doi:10.1016/j.ijpddr.2020.08.005)
Supplement: Supplementary Table S2 — Overview of body weight development (in grams) of piglets in different groups with standard deviations in brackets. SD: study day, BW: body weight, BWG: body weight gain. [file mmc3.docx]

| **Experiment I** | **Groups** | | | |
| --- | --- | --- | --- | --- |
| **Parameter** | **A** | **B** | **C** | **D** |
| Mean AUC for OpG | 4861.8 | 333 | - | 101898 |
| Number of excretion days ± SD | 1 ± 1.56 | 0.45 ± 1.03 | - | 4.4 ± 2.91 |
| Number of diarrhea days ± SD | 1.4 ± 1.71 | 0.27 ± 0.65 | 0.45 ± 0.93 | 3.1 ± 2.23 |
| % piglets with diarrhea at least once | 60 | 18.18 | 27.27 | 90 |
| **Experiment II** | **Groups** | | | |
| **Parameter** | **W** | **X** | **Y** | **Z** |
| Mean AUC for OpG | 99.9 | 20129.85 | 56610 | 139417 |
| Number of excretion days ± SD | 0.1 ± 0.32 | 1.3 ± 2.75 | 3.6 ± 1.94 | 3.3 ± 1.50 |
| Number of diarrhea days ± SD | 0.2 ± 0.63 | 0.5 ± 0.70 | 2.6 ± 1.88 | 4.3 ± 2.39 |
| % piglets with diarrhea at least once | 10 | 40 | 77.80 | 88.90 |
