# Supplementary material for: Reduced treatment frequencies with bumped kinase inhibitor 1369 are effective against porcine cystoisosporosis
Source: Int J Parasitol Drugs Drug Resist. 2020 Aug 21;14:37–45. doi: 10.1016/j.ijpddr.2020.08.005 (PMC7442133; doi:10.1016/j.ijpddr.2020.08.005)
Supplement: Supplementary Table S3 — Limit of Quantitation (LoQ) for BKI 1369 by tissue type. Summary of samples above the LoQ and those that fall between background signal and the LoQ. Tissue samples were taken on study day 29. [file mmc4.docx]

| **Group** | **BW**  **SD 1** | **BW**  **SD 8** | **BW**  **SD 15** | **BW**  **SD 22** | **BW**  **SD 29** | **BWG**  **SD 8 to 15** |
| --- | --- | --- | --- | --- | --- | --- |
| **A** | 1276 [227.4] | 2604 [283.8] | 4198.4 [573.9] | 6082 [750.1] | 7956 [750.22] | 1594.4 |
| **B** | 1214.5 [206.9] | 2530.9 [362.3] | 4292.7 [724.9] | 5892.7 [1225.6] | 7721.8 [1710.2] | 1761.8 |
| **C** | 1163.6 [205.5] | 2494.5 [328.9] | 4267.3 [491.6] | 5950.9 [619.6] | 7867.3 [851.9] | 1772.7 |
| **D** | 1270.0 [165.3] | 2510.0 [285.9] | 3552.0 [434.0] | 5144.0 [506.6] | 7014.0 [547.9] | 1042.0 |
| **W** | 1606.0 [347.4] | 3148.0 [610.4] | 5140.0 [830.8] | 6952.0 [1204.0] | 9242.0 [1250.4] | 1992.0 |
| **X** | 1472.0 [353.0] | 1968.0 [632.7] | 4606.0 [745.5] | 6502.0 [1068.4] | 8752.0 [2465.6] | 1638.0 |
| **Y** | 1444.4 [391.1] | 2771.1 [654.5] | 3891.1 [990.8] | 5782.2 [1340.1] | 8055.6 [1626.8] | 1120.0 |
| **Z** | 1371.1 [331.2] | 2397.8 [643.6] | 2786.7 [962.5] | 4022.2 [1618.8] | 5840.0 [2055.8] | 388.9 |
