# Supplementary material for: Reduced treatment frequencies with bumped kinase inhibitor 1369 are effective against porcine cystoisosporosis
Source: Int J Parasitol Drugs Drug Resist. 2020 Aug 21;14:37–45. doi: 10.1016/j.ijpddr.2020.08.005 (PMC7442133; doi:10.1016/j.ijpddr.2020.08.005)
Supplement: Supplementary File S1 — Compound detection and quantification by LC-MS/MS. [file mmc5.docx]

|  | **LoQ (μM)** | | |
| --- | --- | --- | --- |
| **Tissues** | **BKI 1369** | **BKI 1318** | **BKI 1817** |
| Liver | 0.02 | 0.08 | 0.04 |
| Kidney | 0.04 | 0.08 | 0.02 |
| Muscle | 0.08 | 0.08 | 0.08 |
| Fat | 0.08 | 0.08 | 0.08 |
| Jejunum | 0.08 | 0.20 | 0.08 |
| -Samples above LoQ BKI 1369: muscle (0.09 μM) from piglet 302  -Samples above background, but below LoQ BKI 1369: fat from piglet 302 and jejunum from piglets 302 and 306 | | | |
| -Samples above LoQ BKI 1318: N/A  -Samples above background, but below LoQ BKI 1318: liver from piglet 304 and muscle from piglet 302 | | | |
| -Samples above LoQ BKI 1817: muscle (0.17 μM) and jejunum (0.11 μM) from piglet 302  -Samples above background, but below LoQ BKI 1817: N/A | | | |
